# A TBX5 3′UTR variant increases the risk of congenital heart disease in the Han Chinese population

**DOI:** 10.1038/celldisc.2017.26

**Published:** 2017-07-25

**Authors:** Feng Wang, Dong Liu, Ran-Ran Zhang, Li-Wei Yu, Jian-Yuan Zhao, Xue-Yan Yang, Song-Shan Jiang, Duan Ma, Bin Qiao, Feng Zhang, Li Jin, Yong-Hao Gui, Hong-Yan Wang

**Affiliations:** 1 Children’s Hospital, State Key Laboratory of Genetic Engineering at School of Life Sciences, Institute of Reproduction & Development, Fudan University, Shanghai, China; 2 Co-innovation Center of Neuroregeneration, Key Laboratory of Neuroregeneration of Jiangsu and Ministry of Education, Nantong University, Nantong, China; 3 The State Key Laboratory of Genetic Engineering, Collaborative Innovation Center of Genetics and Development, School of Life Sciences, Fudan University, Shanghai, China; 4 The State Key laboratory for Biocontrol and MOE Key Laboratory of Gene Engineering, School of Life Sciences, Sun Yat-Sen University, Guangzhou, China; 5 Key Laboratory of Molecular Medicine, Ministry of Education, Department of Biochemistry and Molecular Biology, Institute of Medical Sciences, Shanghai Medical College, Fudan University, Shanghai, China; 6 Institute of Cardiovascular Disease, General Hospital of Jinan Military Region, Jinan, China; 7 The Obstetrics & Gynecology Hospital, Key Laboratory of Reproduction Regulation of NPFPC, Institute of Reproduction & Development, Fudan University, Shanghai, China

**Keywords:** congenital heart disease, *TBX5*, variant, 3′UTR, microRNAs

## Abstract

TBX5 is a vital transcription factor involved in cardiac development in a dosage-dependent manner. But little is known about the potential association of *TBX5* 3′ untranslated region (UTR) variations with congenital cardiac malformations. This study aimed to investigate the relationship between *TBX5* 3′UTR variants and risk for congenital heart disease (CHD) susceptibility in two Han Chinese populations, and to reveal its molecular mechanism. The relationship between *TBX5* 3′UTR variants and CHD susceptibility was examined in 1 177 CHD patients and 990 healthy controls in two independent case–control studies. Variant rs6489956 C>T was found to be associated with increased CHD susceptibility in both cohorts. The combined CHD risk for the CT and TT genotype carriers was 1.83 times higher than that of CC genotype, while the risk for CT or TT genotype was 1.94 times and 2.31 times higher than that of CC carriers, respectively. Quantitative real-time PCR and western blot analysis showed that T allele carriers exhibited reduced *TBX5* mRNA and protein levels in CHDs tissues. Compared with C allele, T allele showed increased binding affinity to miR-9 and miR-30a in both luciferase assays and surface plasmon resonance analysis. Functional analysis confirmed that miR-9 and miR-30a downregulated *TBX5* expression at the transcriptional and translational levels, respectively. The assays in zebrafish model were in support of the interaction of miR-9/30a and TBX5 3′UTR (C and T allele). We concluded that *TBX5* 3′UTR variant rs6489956 increased susceptibility of CHD in the Han Chinese population because it changes the binding affinity of two target miRNAs that specifically mediate *TBX5* expression.

## Introduction

Congenital heart diseases (CHDs) are the most prevalent type of human structural birth defects worldwide, affecting between 19 and 75 per thousand live births [1]. The etiology of CHDs includes both genetic and environmental factors. Extensive model organism studies and linkage analysis of rare Mendelian CHD families have provided insights into the genetic basis of CHD, which results from alterations in transcription factors and genes involved in cardiac development [2,3,4,5]. However, mutations in these genes can only explain a small fraction of CHD cases. The pathogenesis of sporadic cases, the most common form of CHD, remains poorly understood.

Genome-wide association studies (GWASs) suggest that a large number of non-coding variants contribute to the risk of various diseases, including cardiovascular disease [6,7,8, 9,10]. Our previous study demonstrated that non-coding variants in genes coding for enzymes that biotransform homocysteine significantly increase CHD risk by regulating gene expression levels [11,12,13]. During embryonic cardiogenesis, many transcription factors including TBX5 [14], GATA4 [15] and NKX2–5 [16] are strictly regulated in a dosage-dependent manner. Mutations in the coding region of *TBX5* invariably result in Holt-Oram syndrome (HOS), an inherited disease characterized by upper limb and cardiac deformities [2, 17]. Thus, we hypothesized that the functional regulatory variations in *TBX5* might be associated with increased CHD risk through altered gene expression or dosage effects.

TBX5 is a transcription factor with well-defined roles in heart and forelimb development. Multiple studies in animal models have confirmed that cardiac development is sensitive to Tbx5 dosage [14, 18, 19]. Most notably, CHD might result from the haploinsufficiency of Tbx5.

Recently, several non-coding variants of *TBX5* in the promoter [20], intron [21] and enhancer regions [22] were reported to be associated with CHDs. Although the 3′ untranslated region (3′UTR) is critically important for microRNAs (miRNAs) binding to regulate gene expression, the potential contribution of mutations in the *TBX5* 3′UTR to CHDs remains unknown. Our previous study demonstrated that miR-10a and miR-10b significantly repressed *TBX5* protein levels by targeting its 3′UTR [23]. In present study, we detected a common variant, rs6489956, in the *TBX5* 3′UTR that significantly increases CHD risk in a Han Chinese cohort composed of 1 177 CHD patients and 990 healthy controls. We further investigated the molecular mechanism underlying that *TBX5* 3′UTR variants influence risk for CHD susceptibility.

## Results

### *TBX5* 3′UTR variant rs6489956 significantly increased CHD susceptibility in Han Chinese

In our study, a total of ten variants in *TBX5* 3′UTR were identified. Among them, four variants had minor allele frequency (MAF) >0.05 and were chosen for genotyping in 288 cases and controls of the Shandong group. Among them, the prevalence of variant rs6489956 (c.* 1101C>T) was significantly different between the CHDs and the control subjects. This variant was then selected for further validation in the other two independent case–control cohorts (Supplementary Table S3).

As the homozygous TT genotype was very rare, we applied the dominant model of inheritance to enhance the statistical power of the study, which combined homozygous TT alleles with heterozygous CT alleles to compare with the wild-type CC alleles in the association studies. Logistic regression analyses demonstrated heterozygote CT and homozygote TT subjects had a significantly increased risk of CHD compared with those with the wild-type CC allele in a study of 905 CHDs and 606 controls in the Shandong group (odds ratio (OR)=2.33, 95% confidence interval (CI)=1.70–3.19, *P*=1.65×10^−7^), as well as 272 CHDs and 384 controls in the Shanghai cohort (OR=1.75, 95% CI=1.24–2.46, *P*=0.0013). The combination of these two independent studies showed a 1.83-fold increased CHD risk when the dominant model is used for *TBX5* rs6489956 (*P*=3.62×10^−8^) in a total number of 1 177 CHDs patients and 990 controls (Table 1), with an adjusted OR of 1.94 (95% CI=1.52–2.47, *P*=8.21×10^−8^) and 2.31 (95% CI=1.01–5.30, *P*=0.033) for the CT and TT genotypes, respectively. Therefore, The variant rs6489956 was significantly associated with an increased risk of CHD in two cohorts separately and together. Similarly, the allele analysis showed that carriers of the susceptible T allele exhibited a higher risk of CHD than carriers of the C allele in both cohorts, resulting in a 1.86-fold combined higher susceptibility of CHD (95%CI=1.50–2.30, *P*=2.01×10^−8^). All genotype frequencies were in accordance with the Hardy-Weinberg expectation among the control subjects (*P*>0.05).

### *TBX5* c.*1101C>T was highly related to septation defects

A stratified analysis was performed according to standard CHDs classifications as previously described [1, 24]. It was observed that *TBX5* c.*1101C>T (rs6489956) is related to multiple types of CHDs (Table 2). Notably, the highest statistical significance was observed in 830 patients with septal defects (heterozygous CT with adjusted OR=1.82, 95% CI=1.41–2.37; homozygous TT with adjusted OR=2.28, 95% CI=0.95–5.49; *P*=9.70×10^−6^). In isolated phenotypes, similar statistical significance was noted in atrial septal defect (ASD) cases (*P*=3.67×10^−3^) and ventricular septal defect (VSD) cases (*P*=2.69×10^−5^).

### The *TBX5* c.*1101C>T affected *TBX5* mRNA transcription and translation

**Figure 1:**
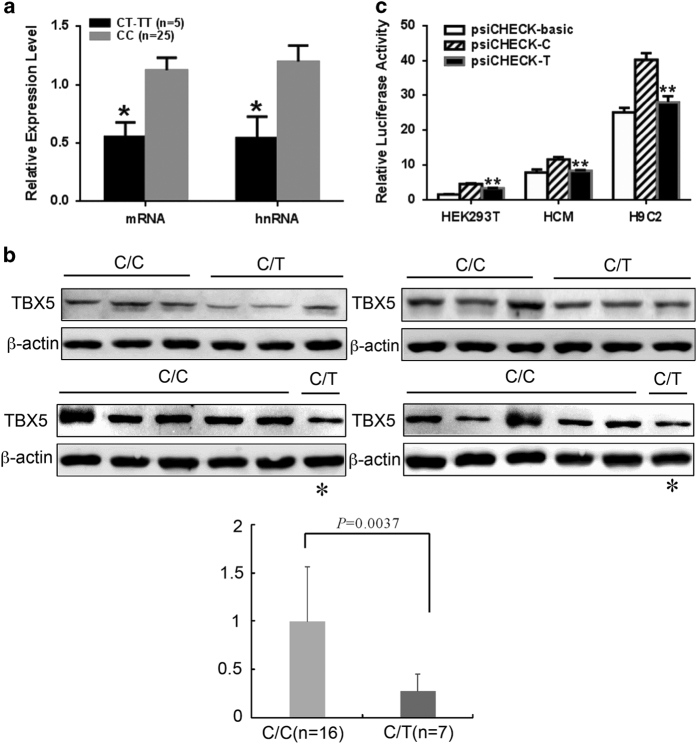
The T allele of rs6489956 reduced *TBX5* mRNA and protein expression levels *in vivo* and *in vitro*. (**a**) Quantitative real-time PCR analysis of TBX5 mRNA levels in 30 cardiac tissue samples from individuals carrying different rs6489956 C>T genotypes. The values for each genotype group were: CC=1.12±0.53 (mRNA), 1.20±0.67 (heterogenous nuclear RNA, hnRNA), CT/TT=0.55±0.27 (mRNA), 0.54±0.41 (hnRNA). All values were normalized to the levels of β-actin and represent the mean±s.d. of three independent experiments. *P<0.05. (**b**) Western Blot analysis in 23 cardiac tissue samples of CHDs indicated that TBX5 protein level in the CT genotype was only 27.1% that of the CC genotype (*P*<0.01). Considering the rare samples of the CT genotype, one of the seven tissues was used twice to compare with the CC genotype and denoted as *. (**c**) Luciferase expression was significantly decreased in the minor T allelic reporter compared with the major C reporter in different cells. The values in HEK 293T cells were: psiCHECK-basic=1.61±1.20, psiCHECK-C=4.60±0.39, psiCHECK-T=3.38±0.22; the values in HCM cells were: psiCHECK-basic=7.85±1.55, psiCHECK-C=11.64±2.03, psiCHECK-T=8.31±0.58; the values in H9C2 cells were: psiCHECK-basic=25.12±3.09, psiCHECK-C=40.18±5.78, psiCHECK-T =28.03±5.35. Each value represented the mean±s.d. of three independent experiments. **P<0.01.

Since the *TBX5* c.*1101C>T (rs648995) variant is located in the *TBX5* 3′UTR, we assumed that this variant might be involved in either *TBX5* transcription or translation. Thus, we examined *TBX5* mRNA expression using quantitative RT-PCR on the cardiac tissue samples. Of the 30 cardiac samples tested, 25 had the rs6489956 CC genotype, four had the CT genotype, and only one had the TT genotype. The relative *TBX5* mRNA expression level in the CT/TT genotypes was only 49.3% of that of the CC genotype (*P*<0.05) (Figure 1a). Consistent with the reduction in mRNA expression, TBX5 protein level in the CT genotype was reduced by 72.9% compared with the CC genotype (0.27±0.19 vs 1.0±0.57, *P*=0.0037) (Figure 1b). Although this study measured *TBX5* mRNA and protein levels in heart tissue samples from subjects who have already been born, which might be different from embryonic *TBX5* levels, our results demonstrated that *TBX5* transcription and translation levels were correlated with the variant rs6489956 genotypes.

Altered *TBX5* mRNA expression associated with the variant rs6489956 was further confirmed by *in vitro* luciferase assays in different cell lines. Consistent with the tissue sample results, the plasmid containing the mutant-T allele showed a 26%, 28% and 31% reduction in luciferase activity when compared with the wild-type C allele in HEK 293T, HCM and H9C2 cells, respectively (*P*<0.01) (Figure 1c). Therefore, both *in vivo* and *in vitro* experimental evidence clearly supports the hypothesis that the *TBX5* c.*1101C>T variant affected the transcription of *TBX5*.

### miR-9 and miR-30a inhibited *TBX5* expression by interacting with the *TBX5* c.*1101C>T variant

**Figure 2:**
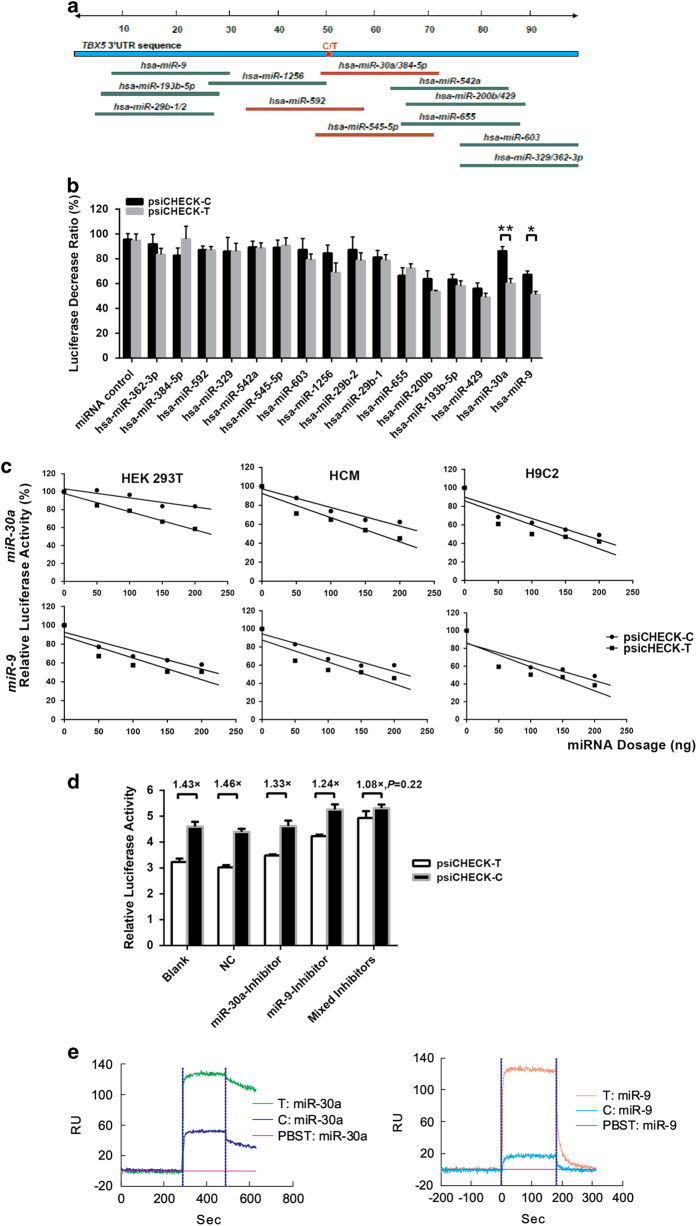
Screen for miRNAs interacting with *TBX5* c.*1101C>T variant. (**a**) 16 Software-predicted miRNAs adjacent to the *TBX5* c.*1101C>T variant; (**b**) the luciferase assay showed that only two miRNAs, miR-30a and miR-9, had statistically significant differences between the two allelic reporters. *P<0.05, **P<0.01. (**c**) Luciferase expression of psiCHECK-C/T was inhibited by miR-30a or miR-9 in a dose-dependent manner, and the inhibition effect was more pronounced with the T allele. (**d**) The expression discrepancy between psiCHECK2-C and T was diminished by miRNA inhibitors, either separately or together; NC represents as negative control. (**e**) SPR analysis results showed the binding affinity of miR-30a or miR-9 to the *TBX5* 3′UTR T allele mRNA was higher than that of the C allele at the same concentration of 20 μm. RU: resonance units; Sec: second.

**Figure 3:**
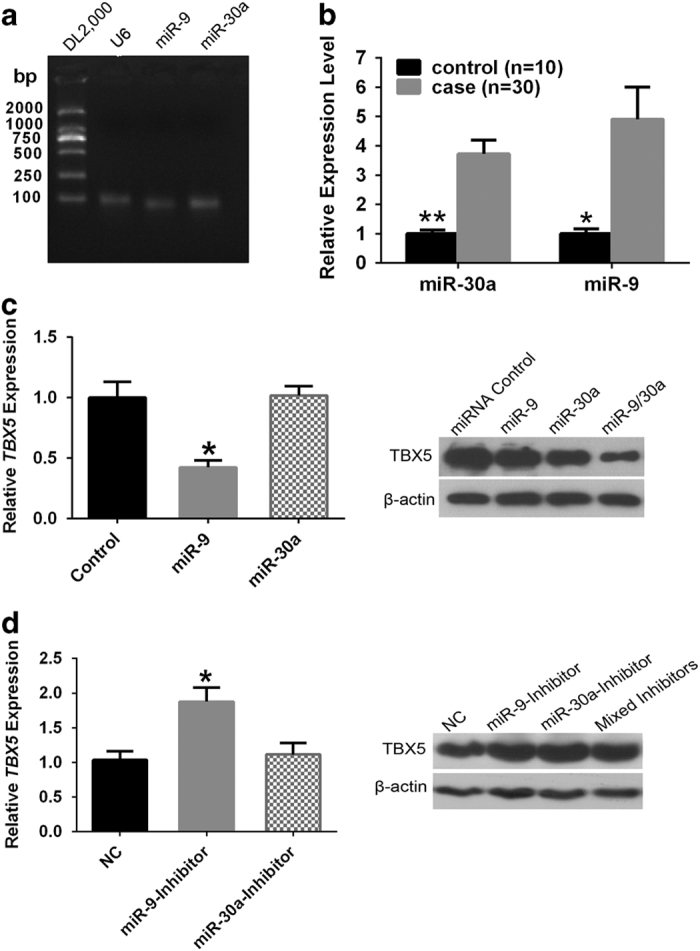
The functional validation of the candidate miRNAs. (**a**) RT-PCR showed that miR-9 and miR-30a were expressed in heart tissues. These tissues originated from the outlet samples of TOF patients during cardiac operation. (**b**) miR-30a and miR-9 expression levels in 30 CHDs heart tissue samples were 3.73 and 4.91 times higher than the controls, respectively. The actual values compared to the internal reference gene levels for each miRNA were as follows: miR-30a=0.096±0.071, miR-9=0.0034±0.0005; (**c**) *TBX5* mRNA expression in HEK 293T cells was downregulated by transfection with miR-9 rather than miR-30a (left), while the presence of both miRNAs inhibited *TBX5* protein concentration according to the western blot analysis results (right). (**d**) *TBX5* mRNA concentration increased in the cells transfected with miR-9 inhibitor, but did not change following transfection of miR-30a inhibitor (left). In contrast, *TBX5* protein concentration was up-regulated with transfection of the two miRNAs inhibitors. NC represents as negative control.

In the flanking region of the *TBX5* 3′UTR variant rs6489956, there are 16 software-predicted miRNAs including five upstream miRNAs, seven downstream miRNAs and four on-site miRNAs (Figure 2a). It was found that only 2 out of these 16 miRNAs, namely, miR-9 and miR-30a, can cause a significant disparity in the expression of the two allelic reporters in HEK 293T cells co-transfected with the psiCHECK-C/T plasmid (Figure 2b). The endogenous expression of miR-9 and miR-30a was detected in all of the 30 human cardiac samples (Figure 3a). We also observed that miR-30a binds to *TBX5* mRNA carrying the T allele with a higher affinity compared with that of the C allele (5.10×10^−7^ moll^−1^[M] vs 1.23×10^−6^ m) (Figure 2e). For the upstream miR-9, although the variant rs6489956 appears not to directly affect miR-9 binding (Supplementary Figure S1), it might impair the miRNAs complex that interacts with the *TBX5* motif containing variant rs6489956, as previously described [25].

The specific inhibition of miR-9 and miR-30a for *TBX5* caused by the 3′ UTR variant c.*1101C>T was demonstrated by a psiCHECK luciferase reporter assay as well as the rescue experiments from miRNA inhibitors. The luciferase gene expression of psiCHECK-C and psiCHECK-T were both inhibited in a linear manner with increased miRNAs concentration, and the miRNAs displayed a more pronounced inhibition effect on psiCHECK-T (Figure 2c). On the other hand, the allelic expression disparity was diminished by the use of each specific inhibitor for miR-9 or miR-30a individually or in tandem (Figure 2d).

### miR-9 and miR-30a inhibited *TBX5* expression at the transcriptional and translational levels independently

It was determined that both miR-9 and miR-30a were present at detectable levels in cardiac tissues (Figure 3a), and the expression of the two miRNAs was 3.7 to 4.9 times higher in cardiac tissues from CHD cases compared with that of cardiac tissues from the non-CHD controls (Figure 3b). The cardiac tissue samples of CHD cases were collected from children (average 4.71 years old), and the non-CHD controls were collected from adults (average 32.7 years old). Despite a potential miRNAs expression disparity between adults and children, such a large difference in miR-9 and miR-30a expression might still represent the potential contributions of the two miRNAs in CHD development, which would require more robust studies to confirm in the future.

Because miR-9 and miR-30a had similar inhibition patterns on the luciferase expression vector with the *TBX5* 3′UTR (Supplementary Figure S2), we measured whether *TBX5* transcription or translation in cultured HEK 293T cells could be regulated by miR-9 and miR-30a. MiR-9 reduced the transcription of *TBX5* mRNA by 57% (Figure 3c, left), and the miR-9 inhibitor up-regulated *TBX5* mRNA expression 1.88-fold (Figure 3d, left). However, neither miR-30a nor its inhibitor had any effect on *TBX5* mRNA expression (Figure 3c and d, left). Both miR-9 and miR-30a could decrease *TBX5* translation (Figure 3c, right). Additionally, miR-9 or miR-30a inhibitors could increase TBX5 protein expression separately or together (Figure 3d, right). These results suggested that miR-9 primarily regulates endogenous transcription of *TBX5*, and that miR-30a regulates translation of the *TBX5* mRNA. Therefore, the mechanisms by which miR-9 and miR-30a regulate *TBX5* were confirmed both *in vitro* and *in vivo*.

### *TBX5* 3′UTR variant rs6489956 interacted with miR-30a or miR-9 in zebrafish

**Figure 4:**
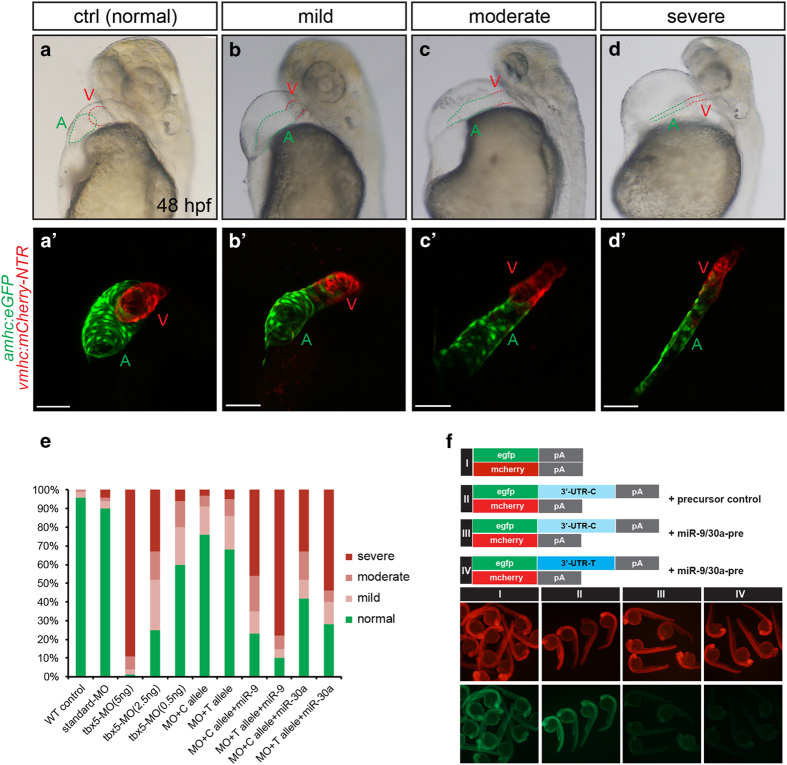
*TBX5* 3′UTR variant rs6489956 affected heart development in zebrafish embryo. (**a**–**d′**) Examples of heart defects with variable severity in zebrafish: (**a** and **a′**) normal heart; (**b** and **b′**) mild defect: heart shows mild looping arrested, pericardium effusion, and dropsy of ventral position; (**c** and **c′**) moderate defect: heart shows moderate looping arrested, pericardium effusion and dropsy of ventral position; (**d** and **d′**) severe defect: malformed embryo with small, string-like heart, severe pericardium effusion, and dropsy of ventral position. Green A indicates ventricle; red V indicates atrium. (**e**) Distribution of four categories of heart morphologies in each experimental group at 48 hpf: WT control, MO standard control, MO (5 ng), MO (2.5 ng), MO (0.5 ng), MO+C allele mRNA, MO+T allele mRNA, MO+C allele mRNA+miR-9, MO+T allele mRNA+miR-9, MO+C allele mRNA+miR-30a, MO+T allele mRNA+miR-30a. (**f**) EGFP sensors were coinjected with mCherry control as indicated. miR-9/30a precursor injection reduced EGFP levels in EGFP-3′UTR sensor (third column), while mCherry levels were unchanged. In the EGFP-3′UTR-T sensor (fourth column), more significant reduction in EGFP was noted.

In order to test the interaction effect of *TBX5* 3′UTR variant and miR-30a or miR-9 *in vivo*, we explored their function on heart development using zebrafish model system. We set up 11 groups for microinjection and did imaging analysis at 48 h post fertilization (hpf): wild-type (WT) control, standard MO control (5 ng), *tbx5* MO (5 ng), *tbx5* MO (2.5 ng), *tbx5* MO (0.5 ng), MO (5 ng)+C allele mRNA (100 pg), MO (5 ng)+T allele mRNA (100 pg), MO (5 ng)+C allele mRNA (100 pg)+hsa-mi9 (100 pg), MO (5 ng)+T allele mRNA (100 pg)+hsa-miR-9 (100 pg), MO (5 ng)+C allele mRNA (100 pg)+hsa-miR-30a (100 pg) and MO (5 ng)+T allele mRNA (100 pg)+hsa-miR-30a (100 pg). As previously reported [26,27,28,29], *tbx5* loss-of-function in zebrafish caused heart developmental defects, including different degrees of heart looping arrested, heart edematous, string-like heart, and so on, which were confirmed in our results (Figure 4a–d′). The *TBX5* 3′UTR variant injection displayed partial rescue of loss-of-function effects in live embryos (Figure 4e). While co-injection of *TBX5* 3′UTR variant with miR-30a or miR-9 into *tbx5* loss-of-function embryos significantly reduced the rescue effects, especially in T allele and miR-30a or miR-9 coinjected embryos (Figure 4e). The fluorescence sensor assay in zebrafish embryos demonstrated that the miR-9/30a precursor repressed the expression of egfp-tbx5-3′-UTR-C and more apparently the expression of egfp-tbx5-3′-UTR-T *in vivo* (Figure 4f). The assays in zebrafish model were in support of the interaction of miR-9/30a and TBX5 3′UTR (C and T allele).

## Discussion

It is well known that the mutations in *TBX5* gene coding regions have a role in syndromic CHD such as Holt-Oram syndrome [2, 30,31,32, 33,34,35,36]. However, the role of *TBX5* in sporadic isolated cases of CHD has not been extensively explored. Experimental evidence based on animal models has confirmed that *Tbx5* has vital roles in cardiac development, and that cardiac development is highly sensitive to *Tbx5* dosage [14, 18, 37, 38]. Therefore, we believed that functional regulatory variants of *TBX5* might contribute to the etiology of sporadic isolated CHDs through a dosage effect. In this study, we investigated all common SNPs in the *TBX5* 3′UTR region and focused on the potential functional variant that we thought might modulate *TBX5* expression by its interaction with miRNAs. The functional variant *TBX5* c.*1101C>T (rs6489956) was found to significantly increase CHD risk in Han Chinese by interacting with miR-30a and miR-9 (OR=1.83. 95% CI=1.48–2.28, *P*=3.62×10^−8^ in the dominant model). Although we first postulated that a functional variant in the *TBX5* 3′UTR may increase the risk of CHD, a recent study indirectly supported our findings by reporting that a *TBX5* enhancer variant causes isolated congenital heart disease [22]. Because our study was only conducted among the Han Chinese subjects, such relationships between variants in the *TBX5* 3′UTR and the risk of CHD need further exploration in other ethnic groups. However, our study elucidated that the sporadic CHDs could be attributed to the combined contribution of the common regulatory variations in the *TBX5* gene, as well as the epigenetic factors such as miR-30a or miR-9.

The possible mechanism of underlying the association of *TBX5* non-coding variants with increased CHD risk may be explained by the sensitive nature of the dosage effect of *TBX5* on heart development. While severe loss-of-function mutations in *TBX5* coding regions led to both heart and forelimb phenotypes, non-coding variants were associated with compartmentalized phenotypes, which might reflect the differing sensitivities of the impacted tissues to the altered *TBX5* expression level. This contention is supported by the fact that the *TBX5* intronic variant rs7312625 was reported to be associated with altered PR intervals (intervals between P wave and R wave in electrocardiogram). In African Americans, and intronic variant rs3825214 was shown to reduce lone atrial fibrillation risk in Han Chinese [39, 40]. The tissue-specific regulation of different non-coding regions could be one possible explanation.

In both embryonic and adult human heart tissues, *TBX5* expression can be observed throughout the epicardium and myocardium, including the free walls and septa of all four cardiac chambers, while its expression in the endocardium is limited to the left ventricle [41]. However, in mouse [42] and chicken [43], *Tbx5* mRNA expression is restricted to the atria and left ventricle. Consistent with these expression patterns, HOS patients with *TBX5* mutations may have a number of significant heart defects, such as septal defects and certain complex malformations (tetralogy of Fallot (TOF), APVR and hypoplastic left heart) [42, 44]. In our study of sporadic CHD cases, we observed that rs6489956 is correlated with a series of cardiac abnormalities; septal defects were statistically most common (*n*=830, *P*=9.70×10^−6^). Additionally, we speculated that the failure to detect any correlation between variant rs6489956 and APVR and outflow tract malformation susceptibility might be due to the limited and insufficient sample size.

Both *in vivo* and *in vitro* assays on the function of variant rs6489956 indicated that the T allele led to reduction in *TBX5* gene expression. We further identified two miRNAs, miR-9 and miR-30a, that interacted with this variant. This is not surprising, because miRNAs are predicted to function in the post-transcriptional regulation of nearly one third of all human genes [45]. It was confirmed that miR-30a did not affect the concentration of *TBX5* mRNA, but reduced its protein concentration, while miR-9 downregulated both transcription and translation of *TBX5*. It has been extensively reported that miRNAs help to regulate cardiac growth and remodeling, and contribute to specific myocyte properties. miRNAs are implicated in numerous cardiovascular diseases, such as heart hypertrophy, heart failure, atherosclerosis, acute myocardial infarction, VSD, TOF and arrhythmias [46–52]. Thus, when the expression of the developmentally important transcription factor, *TBX5* was disturbed during a sensitive time window of development, it increases the likelihood of inducing CHD. As a new model trigger, the interactions between miRNAs and *TBX5* regulatory variant increase the CHD risk through the downregulated *TBX5*, either at the post-transcriptional or translational level.

Because miRNAs are vital factors that mainly target 3′UTR sequences of mRNA, we suspected that miRNAs might have important roles in CHDs. Both bioinformatic predictions and functional analyses showed that both miR-30a and miR-9 have a higher affinity to the minor T allele. We further confirmed these results using luciferase assays, quantitative RT-PCR, western blot analysis and whole-embryo microRNA sensor assay in zebrafish. Recently, many studies have implicated miRNAs in various cardiovascular diseases. For example, miRNA-1–2 knockout mice have decreased levels of the cardiac transcription factor Hand2, which results in heart malformations (VSD) similar to those observed in *Hand2*-deficient mice [53, 54]. Comparisons of malformed heart tissues from CHD patients with healthy controls have demonstrated dysregulation of miRNAs. However, to our knowledge, no evidence has shown that variations of miRNAs sequence, DNA binding motif or even altered miRNAs levels can directly cause CHD. Our study initially demonstrated that certain miRNAs can inhibit the expression of important transcription factors for cardiac development through the alternative binding sites and variable binding affinities, which eventually increase CHD risk. We also found that the levels of miR-30a and miR-9 were 3.72 and 4.91 times higher in CHD children compared with non-CHD adult controls. If it is true that miR-30a and miR-9 expression level changes are minimal after the heart has formed, our data suggest that miR-30a and miR-9 are likely contributors to the occurrence of CHDs. Because miRNAs are surprisingly stable and detectable in the circulating blood stream, they could serve as novel biomarkers for molecular diagnostics, as well as the promising therapeutic targets after they are validated by further studies. Therefore, future investigations on the interaction of CHD-associated regulatory variants in the master transcription factors and its target miRNAs will open a door for the improved CHD diagnosis and therapy.

In summary, our study demonstrated a significant effect of *TBX5* 3′UTR variant rs6489956 on the increased susceptibility of CHD in two independent Han Chinese populations. It was well known that both *Tbx5* overexpression and insufficiency result in heart defects in developing embryos. In view of the decreased *TBX5* mRNA levels detected in the heart tissues of CHD patients with the CT and TT genotypes, the individuals with the T allele are susceptible to develop CHD due to the *TXB5* deficiency. Functional studies clearly showed that the consequent repression of *TBX5* expression on the post-transcriptional level resulted from an enhanced binding affinity of the T allele to miRNAs. Our data highlighted an important function of the underlying genetic variations in the 3′UTR of *TBX5* in CHDs, providing new insight into risk assessment for these common birth defects.

## Materials and Methods

### Study subjects

We analyzed samples from two independent case–control groups. The Shandong group consisted of 905 CHD patients and 606 matched controls recruited from the Cardiovascular Disease Institute, General Hospital of Jinan Military Command (Jinan, Shandong Province, China) between August 2009 and September 2011. The Shanghai group was composed of 272 CHD cases and 384 matched controls enrolled in the Children’s Hospital of Fudan University (Shanghai, China) between August 2010 and November 2011. All subjects were unrelated ethnic Han Chinese. All of the controls were non-CHD outpatients from the same geographical area who were matched to the affected individuals in terms of age and sex over the same period. CHDs patients who were considered to be syndromic and not isolated CHDs, or who had a positive family history of CHD in a first-degree relative (parents, siblings and children), were excluded from the study.

There were no newborn samples in our study. We classified the 1 177 CHDs cases into seven broad categories according to the commonly accepted criteria [24]. Specifically, 156 (13.3%) had conotruncal defects, 830 (70.5%) had septal defects, 17 (1.4%) had left ventricular outflow tract obstruction (LVOTO), 24 (2.0%) had right ventricular outflow tract obstruction (RVOTO), 16 (1.4%) had anomalous pulmonary venous return (APVR), 14 (1.2%) had complex CHD and 120 (10.2%) had other cardiac abnormalities (Supplementary Table S1).

To screen for genetic variants in the 3′UTR of *TBX5* gene, we selected a subset of 32 subjects for resequencing. Half of these subjects were CHD patients, and half were controls. Fifty-three cardiovascular tissue samples were obtained with informed consent from CHD patients who had undergone cardiac operations from January 2011 to September 2011 in the Cardiovascular Disease Institute, General Hospital of Jinan Military Command (Jinan, Shandong Province, China) and Children’s Hospital of Fudan University (Shanghai, China), among which 30 were used for quantitative real-time PCR (RT-PCR) assay and 23 for western blot analysis. The 53 collected cardiac tissue samples comprised 10 atrium samples, 4 ventricle samples, 4 appendage sample, 8 atrial septum samples, 4 ventricular septum samples, 15 outlet samples, and 8 aorta samples. Normal heart tissue samples were collected by the Department of Forensic Medicine, Fudan University from adults with no history of heart disease who died of accidents.

All study protocols were reviewed and approved by the medical ethics committee of Children’s Hospital of Fudan University, and written consent was obtained from the parents of subjects and/or the subjects themselves before study enrollment.

### Variant identification and genotyping

Genomic DNA was isolated from the peripheral venous blood using conventional regents. *TBX5* 3′UTR fragments were amplified using PCR to amplify a 1757-bp fragment from 114793401 to 114791645 (NM_000192) in 32 unrelated individuals randomly selected for variant screening by sequencing. Direct dye terminator sequencing of the PCR products was carried out using the ABI Prism BigDye system according to the manufacturer’s instructions (Applied Biosystems, Foster City, CA, USA). Selected single-nucleotide polymorphisms (SNPs) with minor allele frequency (MAF) >0.05 were genotyped using SNaPshot analysis (ABI). In the first stage of the present study, a total of 288 CHD cases and the same number of controls of the Shandong group were collected and genotyped in October 2010. The variants associated with CHD risk were chosen for further case–control study in the following Shandong and the Shanghai samples. The samples were sequenced and genotyped on an ABI 3730 automated sequencer and analyzed using Lasergene 7.0 and Peakscanner 1.0, respectively. All of the PCR and sequencing primers were listed in Supplementary Table S2.

### Quantitative real-time PCR

Total RNA was extracted from human cardiovascular tissue samples preserved in RNAlater (Qiagen, Valencia, CA, USA) and converted to cDNA using random hexamers, oligo(dT) primers and PrimeScript RT reagent Kit (TaKaRa, Tokyo, Japan). Quantitative Real-time PCR was performed with SYBR Premix Ex Taq (TaKaRa) using a StepOne Real-Time PCR system (ABI) with β-actin as an internal reference gene. Each reaction was performed in triplicate. The primers were described in Supplementary Table S2.

### Western blot analysis

Proteins were separated on 10% sodium dodecyl sulfate polyacrylamide electrophoresis gels and blotted on to polyvinylidene fluoride membranes (Millipore, Billerica, MA, USA). Non-specific binding was blocked with 5% skimmed milk in TBS. Blots were probed with either mouse horseradish peroxidase (HRP)-conjugated anti-β-actin monoclonal antibody (Kangchen Bio-tech, Shanghai, China), or rabbit anti-TBX5 polyclonal antibody (Abcam, Cambridge, UK) followed by HRP-conjugated goat anti-rabbit secondary antibody (Proteintech, Chicago, IL, USA). Proteins were detected using ECL reagents (Millipore).

### Plasmid

To construct the reporter plasmids, *TBX5* 3′UTR containing either the C or T allele was amplified from genomic DNA and subcloned into the downstream region of Renilla luciferase using *Xho*I and *Not*I restriction sites in psiCHECK-2 vector (Promega, Madison, WI, USA). Primers were listed in Supplementary Table S2. All recombinant clones were verified by DNA sequencing.

Human wild-type *TBX5* ORF fused to PCMV6-XL4 was purchased from Origene Technologies (Beijing, China). The mutant *TBX5* was generated by the the Quick Change Site-Directed Mutagenesis Kit (Stratagene). Constructs were linearized using *Pvu*II and the capped and polyA tailed mRNA was synthesized with mMessage mMACHINE T7 Ultra Kit (Ambion, Life Technologies) for the zebrafish microinjection.

### Cell culture and luciferase reporter assays

Human embryonic kidney 293T (HEK 293T), human cardiac myocytes (HCM) and rat cardiac myocyte (H9C2) cells were grown in Dulbecco’s Modified Eagle’s Medium supplemented with 10% fetal bovine serum.

HEK 293T, HCM or H9C2 cells were seeded in 24-well culture plates at the density of 4.0×10^5^, 2.0×10^5^ and 1.0×10^5^ per liter, respectively. Twenty-four hours later, the cells were transfected with 400 ng of each *TBX5* reporter plasmid using Lipofectamine 2000 (Invitrogen, Carlsbad, CA, USA). To screen the predicted candidate miRNAs, HEK 293T cells were co-transfected with 25 ng of psiCHECK-C/T plasmid and 75 ng of miRNA expression vector in 96-well culture plates. To further explore the interaction between *TBX5* 3′UTR and selected candidate miRNAs, HEK 293T, HCM or H9C2 cells were co-transfected with 50 ng psiCHECK-C/T plasmid as well as 0 ng, 50 ng, 100 ng, 150 ng or 200 ng of miRNA expression vector. For small interfering RNA assays, HEK 293T cells were co-transfected with 100 ng psiCHECK-C/T plasmid and 100 nm miRNA inhibitors (RiboBio, Guangzhou, China) in 24-well culture plates.

Cells were lysed 24 h after transfection for luciferase assays that employed the Dual Luciferase Reporter Assay System (Promega) according to the manufacturer’s instructions. Three independent transfection experiments were performed, and each luciferase assay was conducted in triplicate.

### Validation of miRNA regulation

HEK 293T cells were transfected with 2 μg of miRNA expression vector or 100 nm of miRNA inhibitors in 6-well culture plates. Cells were harvested and lysed 48 h after transfection in RIPA buffer and assayed for the relative protein concentrations of intracellular TBX5 (rabbit anti-TBX5 polyclonal antibody; Abcam) and β-actin (Kangchen Bio-tech) using western blot analysis. RNA was also purified from these cells for quantitative RT-PCR analysis of *TBX5* mRNA levels.

### Surface plasmon resonance

SPR analysis was conducted using the ProteOn XPR36 protein interaction array system (Bio-Rad, Hercules, CA, USA). Biotinylated miRNAs were immobilized to an individual channel of the streptavidin-modified sensor chip at a fixed concentration of 400 nm. Single-stranded RNA (GenePharma, Shanghai, China) harboring 32 bp of 3′UTR wild-type-C allele RNA or 3′UTR mutant-T-allele was diluted into a variety of concentrations in PBST buffer (10 mm Na_2_HPO_4_, 150 mm NaCl and 0.005% Tween 20, pH 7.4) and flowed over the biotinylated miRNAs. At the end of each cycle, 5 mm NaOH was applied to regenerate the sensor surface. Data were converted by BIA evaluation software.

### Zebrafish, microinjection and imaging

The morpholino (MO) antisense oligonucleotide MO-Tbx5a (5′- GAAAGGTGTCTTCACTGTCCGCCAT-3′) was purchased from Gene Tools (Philomatch, OR, USA). Human miR-30a, mir-9 and a negative control were purchased from Thermo Fisher Scientific (Waltham, MA, USA). Zebrafish were raised under standard conditions at 28.5 °C. Each 1-2-cell stage embryo was injected with a constant injection of 5 ng MO, 100 pg *TBX5* mRNA and 100 pg miRNAs using a microinjector (Narishige, Japan). Twelve hours post-injection, the dead embryos were removed, leaving only viable embryos that were used for further analysis. Consistent with the previously published studies [18, 55], all live embryos were divided into the four categories according to their heart morphologies. At 48-h post fertilization (hpf), images were acquired with an Olympus stereomicroscope microscope or Leica TCS-SP5 LSM confocal microscope. For confocal imaging analysis of zebrafish embryos, they were anesthetized with egg water/0.16 mgml^−1^ tricaine/1% 1- phenyl-2-thiourea (Sigma-Aldrich, St Louis, MO, USA) and embedded in 0.6% low melting agarose. Confocal imaging analysis was performed using Imaris software. Two transgenic zebrafish lines: *Tg(vmhc:eGFP)* and *Tg(vmhc:mCherry-NTR)* were used as described in previous work [55]. Whole-embryo microRNA sensor assay in zebrafish was carried out as described previously [56].

### Statistical analysis

The Hardy-Weinberg equilibrium test was performed on controls using the *χ*^2^-test. Differences in demographic features and allelic or genotypic frequencies between the CHD cases and controls were evaluated using Pearson’s *χ*^2^-test. To evaluate associations between the genotypes and CHD risk, ORs and 95% CIs were calculated by unconditional logistic regression analysis with adjustments for age and sex. In multiple comparison testing, we employed the Bonferroni correction. The significance level was adjusted to *P*=0.0025 given the four SNPs being tested for 5 genetic models in 288 cases and the same number of controls.

Data were calculated as the mean±s.d. Significant differences between the test and control groups were analyzed by means of an independent *t*-test with Bonferroni correction. The *P*<0.05 was defined as statistical significance. Statistical analyses were conducted with SPSS 16.0 software (SPSS, Chicago, IL, USA).

## Figures and Tables

**Table 1:** Association between rs6489956 in the 3′UTR region of *TBX5* and risk of congenital heart disease in two independent case–control studies

*Group*	*Genotype/Allele*	*Control*	*Case*	*Logistic regression*		*HWEp*^a^
				*OR (95% CI)*	P*-value*^b^	
Shandong	C/C	534(88.1%)	703(77.7%)	1.00	**1.65×10**^**−7**^	0.085
	C/T-T/T	72(11.9%)	202(22.3%)	**2.33 (1.70–3.19)**		
	C	1 135(93.6%)	1 595(88.1%)	1.00	**2.86×10**^**−7**^	
	T	77(6.4%)	215(11.9%)	**2.16 (1.61–2.91)**		
Shanghai	C/C	318(82.8%)	197(72.4%)	1.00	**0.0013**	0.54
	C/T-T/T	66(17.2%)	75(27.6%)	**1.75 (1.24–2.46)**		
	C	698(90.9%)	462(84.9%)	1.00	**0.001**	
	T	70(9.1%)	82(15.1%)	**1.77 (1.26–2.49)**		
Combined	C/C	852(86.1%)	900(76.5%)	1.00	**3.62×10**^**−8**^	0.1
	C/T-T/T	138(13.9%)	277(23.5%)	**1.83 (1.48–2.28)**		
	C	1 833(92.6%)	2 057(87.4%)	1.00	**2.01×10**^**−8**^	
	T	147(7.8%)	297(12.6%)	**1.86 (1.50–2.30)**		

Abbreviations: CI, confidence interval; HWE, Hardy-Weinberg equilibrium; OR, odds ratio.

a *P*-value for HWE test in control subjects.

b Adjusted for age and gender. The bold entries indicate significant P values.

**Table 2:** Stratified analysis of rs6489956 by CHDs classification

	*No.*	P*-value*	*Association (OR (95% CI))*^a^ *CT/TT vs CC*
*CHDs classification I*^b^			
Conotruncal defects	156	**4.63×10**^**−5**^	**2.35 (1.56–3.54)**
Septal defects	830	**1.71×10**^**−6**^	**1.85 (1.44–2.39)**
LVOTO	17	0.171	2.27 (0.70–7.29)
RVOTO	24	0.114	2.14 (0.83–5.52)
APVR	16	0.94	0.94 (0.21–4.25)
Complex CHDs	14	**0.025**	**3.58 (1.17–10.93)**
Other cardiac abnormalities	120	**0.006**	**2.22 (1.26–3.93)**
			
*CHDs classification II*			
Isolated CHDs	1 016	**6.67×10**^**−7**^	**1.85 (1.45–2.36)**
Non-isolated CHDs	161	**9.83×10**^**−7**^	**2.68 (1.81–3.98)**
			
*Detailed CHDs phenotypes*			
ASD	120	**3.67×10**^**−3**^	**2.00 (1.25–3.18)**
VSD	691	**2.69×10**^**−5**^	**1.76 (1.35–2.30)**
TOF	98	**0.031**	**1.78 (1.06–3.02)**

Abbreviations: APVR: anomalous pulmonary venous return; ASD: atrial septal defect; LVOTO: left ventricular outflow tract obstruction; RVOTO: right ventricular outflow tract obstruction; TOF: tetralogy of Fallot; VSD: ventricular septal defect.

a Adjusted for age and gender.

b Classification described in Botto *et al*. [24].The bold entries indicate significant P values.
