# Supplementary material for: A TBX5 3′UTR variant increases the risk of congenital heart disease in the Han Chinese population
Source: Cell Discov. 2017 Jul 25;3:17026–. doi: 10.1038/celldisc.2017.26 (PMC5527299; doi:10.1038/celldisc.2017.26)
Supplement: Supplementary Information [file celldisc201726-s1.docx]

**SUPPLEMENTAL MATERIAL**

**Supplemental Table 1. Demographic characteristics in CHD cases and controls**

|  | **Cases** | | **Controls** | | ***P* value** |
| --- | --- | --- | --- | --- | --- |
|  | **No.** | **%** | **No.** | **%** |  |
| Stage 1, Shandong Group | *N*=905 |  | *N*=606 |  |  |
| Age (years,Mean±SD) | 6.28±4.50 |  | 6.51±3.19 |  | 0.27 |
| Gender |  |  |  |  | 0.67 |
| Male | 461 | 50.9 | 316 | 52.1 |  |
| Female | 444 | 49.1 | 290 | 47.9 |  |
| Stage 2, Shanghai Group | *N*=272 |  | *N*=384 |  |  |
| Age,years(Mean±SD) | 7.34±5.66 |  | 7.24±3.16 |  | 0.81 |
| Gender |  |  |  |  | 0.43 |
| Male | 156 | 57.4 | 207 | 53.9 |  |
| Female | 116 | 42.6 | 177 | 46.1 |  |
| Combined Samples | *N*=1,177 |  | *N*=990 |  |  |
| Age,years(Mean±SD) | 6.55±4.83 |  | 6.82±3.19 |  | 0.14 |
| Gender |  |  |  |  | 0.86 |
| Male | 617 | 52.4 | 523 | 52.8 |  |
| Female | 560 | 47.6 | 467 | 47.2 |  |
| CHD Classification 1 |  |  |  |  |  |
| Conotruncal Defects | 156 | 13.3 |  |  |  |
| Sepation Defects | 830 | 70.5 |  |  |  |
| LVOTO | 17 | 1.4 |  |  |  |
| RVOTO | 24 | 2.0 |  |  |  |
| APVR | 16 | 1.4 |  |  |  |
| Complex CHD | 14 | 1.2 |  |  |  |
| Other Cardiac Abnormalities | 120 | 10.2 |  |  |  |
| CHD Classification 2 |  |  |  |  |  |
| Isolated CHD | 1016 | 86.3 |  |  |  |
| Non-isolated CHD | 161 | 13.7 |  |  |  |
| Detailed Phenotypes |  |  |  |  |  |
| ASD | 120 | 10.2 |  |  |  |
| VSD | 693 | 58.9 |  |  |  |
| TOF | 98 | 8.3 |  |  |  |

All comparisons by t test or two-sided χ2 test

**Supplemental Table 2. DNA or RNA sequence of all used primers**

| Primer Name | Sequence (5’ to 3’) | Assay |
| --- | --- | --- |
| UTR3-F1 | GTTGGCATGGTGCCAGAGTG | PCR/Sequence |
| UTR3-R1 | TGTGGTGGTAGTGGGGGGTG | PCR/Sequence |
| UTR3-F2 | AGAGAACCCCACGGACAAGA | PCR/Sequence |
| UTR3-R2 | CAGACCTCCCCCCAAATAAG | PCR/Sequence |
| UTR3-F3 | CCCTTATTTGGGGGGAGGTCTG | PCR/Sequence |
| UTR3-R3 | CATTGGTGTGGGCGTGGTTTCT | PCR/Sequence |
| UTR3-F4 | AGCCCCAACCTTCCAAACCT | PCR/Sequence |
| UTR3-R4 | AATCCTCACCCTCCCCCCTT | PCR/Sequence |
| UTR3-F5 | GGGGGGAGGGTGAGGATT | PCR/Sequence |
| UTR3-R5 | TGCGATGGGCATGAGAAA | PCR/Sequence |
| UTR3-miltiplex-PCR-F1 | CTCTTCCCCCATTCCTTC | PCR |
| UTR3-miltiplex-PCR-R1 | CTCAGACCTCCCCCCAAA | PCR |
| rs12426660 A>G typing | TAGTGCGTAGTTGGAGTCTG | SNaPshot Genotying |
| rs6489956 C>T typing | tttttACCACGCCCACACCAATGCC | SNaPshot Genotying |
| rs883079 C>T typing | tttttttttttttttGTGAAATGAAAAATCTTGTC | SNaPshot Genotying |
| rs10850326 T>C typing | ttttttttttttttttttttCAGACATTTCCTAGAGAAAG | SNaPshot Genotying |
| *TBX5-*mRNA-F | TCCAGAAACTCAAGCTCACC | qRT-PCR |
| *TBX5-*mRNA-R | TGCTGTCACCTTCACCGTTC | qRT-PCR |
| *ACTB-*mRNA-F | TAGTTGCGTTACACCCTTTCTTG | qRT-PCR |
| *ACTB-*mRNA-R | TGCTGTCACCTTCACCGTTC | qRT-PCR |
| *TBX5*‐nascent‐F | AGGATTTCGGGGCAGTGAT | qRT-PCR |
| *TBX5*‐nascent‐R | AAGGCTGGTGGAGGGAGGT | qRT-PCR |
| *TBX5*-UTR3-XhoⅠ-F | CCGCTCGAG CCCCTCATCAGTACCACTCTGT | Clone Construct |
| *TBX5*-UTR3-NotⅠ-R | ATAAGAATGCGGCCGCAACCTCTTCCTGTTTCCTCCAA | Clone Construct |
| *TBX5*-UTR3-M1101-F | CCAATGCCAACACAAAACTGTGTTTACTG | Point Mutation |
| *TBX5*-UTR3-M1101-R | CAGTAAACACAGTTTTGTGTTGGCATTGG | Point Mutation |
| *TBX* 5-PCMV-Mutant-F | CCCACACCAATGCCGACACAAAACTGTG | Point Mutation |
| *TBX* 5-PCMV- Mutant-R | CACAGTTTTGTGTCGGCATTGGTGTGGG | Point Mutation |
| *TBX5*-UTR3-M1073-F | CACCAATGATATCTCGGGTTTCTAACCACGCCCACACCAAT | Overlap PCR |
| *TBX5*-UTR3-M1073-R | ATTGGTGTGGGCGTGGTTAGAAACCCGAGATATCATTGGTG | Overlap PCR |
| *TBX5*-UTR3-M1113-F | TGCCAACACAAAACTGACAAATGTGAAAGCCGAAAACAG | Overlap PCR |
| *TBX5*-UTR3-M1113-R | CTGTTTTCGGCTTTCACATTTGTCAGTTTTGTGTTGGCA | Overlap PCR |
| UTR3-T-mRNA-mir30a | AUGCCAACACAAAACUGUGUUUACUGAAAGC | SPR |
| UTR3-C-mRNA-mir30a | AUGCCGACACAAAACUGUGUUUACUGAAAGC | SPR |

**Supplemental Table 3. Genotype frequency of four *TBX5* 3’UTR SNPs in 288 CHD patients and 288 controls**

| **SNPs** | **Genotype** | **Control** | **Case** | ***P* value** | **HWE*p**** | **% Genotyped** |
| --- | --- | --- | --- | --- | --- | --- |
|  | A/A | 235(83.3%) | 230(81%) |  |  |  |
| rs12426660 | A/G | 45(16%) | 53(18.7%) | 0.59 | 1 | 98.3 |
|  | G/G | 2(0.7%) | 1(0.4%) |  |  |  |
|  | C/C | 258(90.8%) | 234(81.8%) |  |  |  |
| rs6489956 | C/T | 24(8.4%) | 50(17.5%) | **0.0016** | 0.14 | 99.0 |
|  | T/T | 2(0.7%) | 2(0.7%) |  |  |  |
| rs883079 | C/C | 100(34.7%) | 92(32.1%) |  |  |  |
|  | C/T | 139(48.3%) | 146(50.9%) | 0.78 | 1 | 99.8 |
|  | T/T | 49(17%) | 49(17.1%) |  |  |  |
| rs10850326 | T/T | 128(44.4%) | 109(38%) |  |  |  |
|  | T/C | 126(43.8%) | 141(49.1%) | 0.29 | 0.79 | 99.8 |
|  | C/C | 34(11.8%) | 37(12.9%) |  |  |  |

* *p* value of HWE in control group


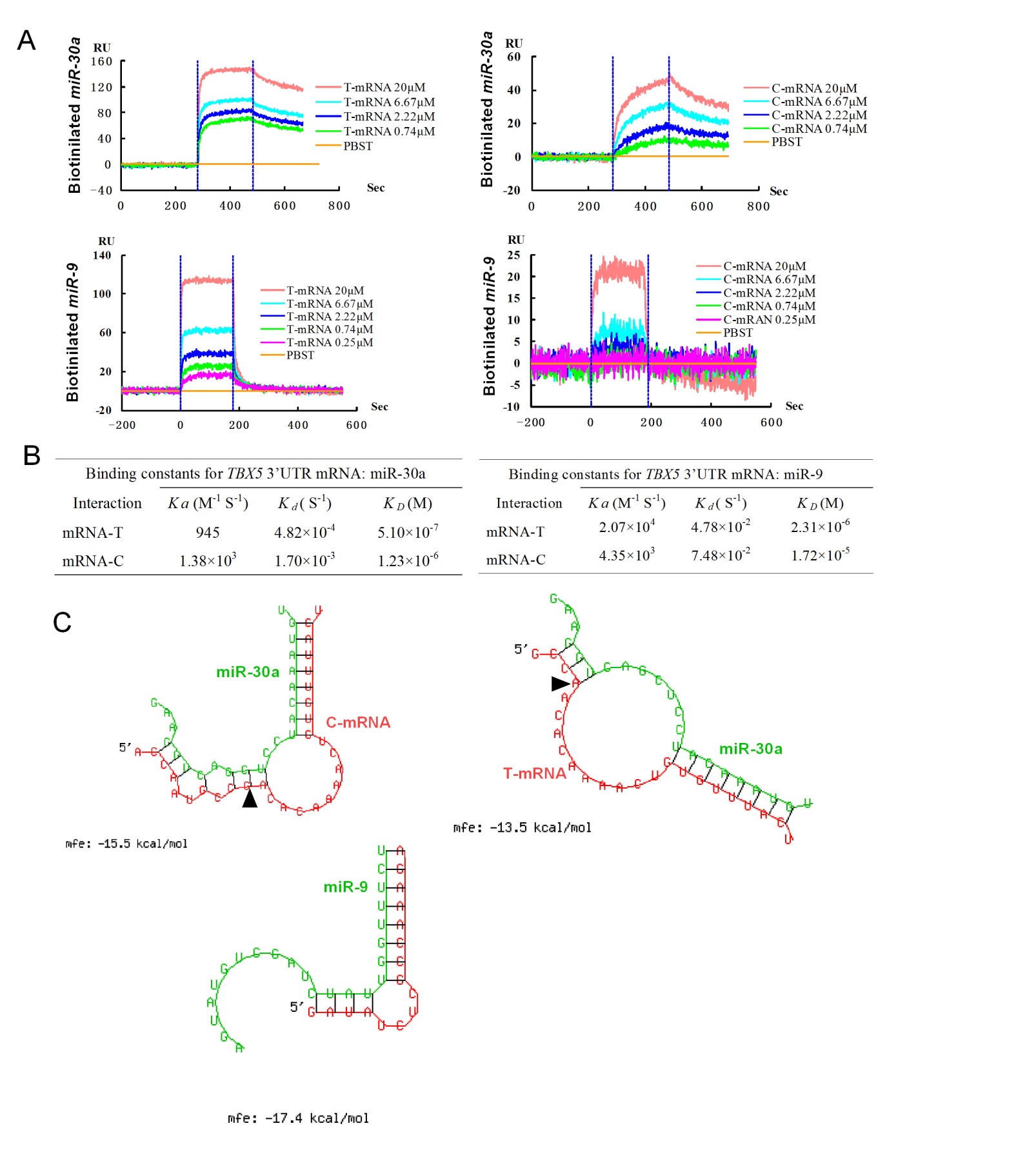


**Supplemental Figure S1 miRNAs binding to mRNA for different allelic 3’UTR of *TBX5* by SPR analysis**

1. Biotin-labeled miR-30a or miR-9 was immobilized to a streptavidin-modified sensor chip. Single-stranded RNA harboring 31-bp *TBX5* 3’UTR wide-type-C-allele RNA or mutant-T-allele RNA was diluted with PBST at different concentrations. The binding signal of the T-allele mRNA interaction with miR30a or miR-9 was much higher than that of C-allele at all of the same concentrations;
2. Data of the binding affinity between mRNA carrying the T or C allele and the corresponding miRNAs were indicated. A stronger binding of mRNA carrying the T-allele was confirmed than that of the C-allele, to miR-30a (5.10×10^-7^ M vs 1.23×10^-6^ M) and miR-9(2.31×10^-6^ M vs 1.72×10^-5^ M), respectively.
3. RNAhybrid prediction of miRNAs binding disparity to different allelic *TBX5* 3’UTR sequence. mfe: minimal free energy

**Supplemental Figure S2 MiR-9 and miR-30a primarily inhibit *TBX5* expression in HEK 293T cells**

(A) *TBX5* Location of predicted target sites for miR-30a and miR-9 in *TBX5* 3’UTR according to TargetScan software. Sequence inspection indicated that *TBX5* 3’UTR contains one binding element for miR-9 and two for miR-30a.

(B) Luciferase assays indicated that both of miR-9 and miR-30a could significantly down-regulate Renilla luciferase gene expression compared to miRNA control (***P*<0.01,* *P*<0.05).
